# Distinct Trajectories of Amygdala Connectivity Patterns Characterize Remission vs. Non‐Remission in Patients With Major Depressive Disorder

**DOI:** 10.1155/da/4701907

**Published:** 2026-06-11

**Authors:** Shudong Zhang, Zhifang Zhang, Xiaoya Li, Jingjing Zhou, Ruinan Li, Rui Liu, Yun Wang, Xiongying Chen, Yuan Feng, Jian Cui, Ling Zhang, Yuan Zhou, Gang Wang

**Affiliations:** ^1^ Beijing Key Laboratory of Intelligent Drug Research and Development for Mental Disorders, National Clinical Research Center for Mental Disorders, National Center for Mental Disorders, Beijing Anding Hospital, Capital Medical University, Beijing, China, ccmu.edu.cn; ^2^ Department of Psychiatry, Shandong Daizhuang Hospital, Jining, China; ^3^ State Key Laboratory of Cognitive Science and Mental Health, Institute of Psychology, Chinese Academy of Sciences, Beijing, China, cas.cn; ^4^ Department of Psychology, University of Chinese Academy of Sciences, Beijing, China, ucas.ac.cn

**Keywords:** amygdala, antidepressant, longitudinal study, major depressive disorder, rest-state functional connectivity

## Abstract

**Background:**

This study aimed to investigate the neural basis of individual differences in antidepressant efficacy using an 8‐week longitudinal multitime point resting‐state functional magnetic resonance imaging (fMRI) design.

**Methods:**

Forty‐eight patients with major depressive disorder (MDD) completed two or three scans, and 44 healthy controls (HCs) underwent a baseline scan. Patients were categorized into remission (MDDr) and nonremission (MDDnr) groups based on treatment outcomes. Group differences in resting‐state functional connectivity (rsFC) of the amygdala subregions at baseline were examined among MDDr, MDDnr, and HCs. Longitudinal changes in the identified rsFC were compared between the MDDr and MDDnr groups. Correlation analyses were conducted to explore the relationship between baseline rsFC or its longitudinal changes and depressive symptom severity or improvement.

**Results:**

At baseline, rsFC between the right basolateral (BL) amygdala and the right supplementary motor area (SMA) was lower in the MDDr group but higher in the MDDnr group compared with HCs, although this effect did not survive multiple comparisons correction across amygdala subregions. The trajectory of this rsFC differed between the two patient groups during treatment, with normalization observed at 2 and 8 weeks posttreatment. Correlation analyses indicated that baseline rsFC was associated with treatment response and that longitudinal changes in rsFC were aligned with symptom improvement, although some associations did not survive multiple comparisons correction.

**Conclusions:**

Our findings provide novel and valuable insights into the neural mechanisms underlying antidepressant response and highlight the role of amygdala subregional connectivity in explaining interindividual variability in treatment efficacy.

**Trial Registration:**

ClinicalTrials.gov identifier: ChiCTR2400093823

## 1. Introduction

Major depressive disorder (MDD) is a prevalent and disabling psychiatric condition that affects affective, cognitive, and somatic domains [1]. Although antidepressants remain the first‐line treatment for MDD [2], their clinical use is challenged by substantial interindividual variability in treatment response [3]. It is estimated that one‐third to one‐half of patients do not achieve significant clinical benefits from antidepressant therapy [4–6]. Therefore, elucidating the neurobiological underpinnings of this heterogeneity is critical for advancing precision medicine in depression.

Recent advances in functional magnetic resonance imaging (fMRI) studies have provided important insights into the effects of antidepressants on intrinsic brain function in patients with MDD [7–11]. A subset of these studies has further examined antidepressant effects by stratifying patients according to treatment outcomes. These findings suggest that patients who achieve remission or exhibit treatment responsiveness can be distinguished from nonresponders or treatment‐resistant individuals after a conventional acute treatment phase (8 or 12 weeks) based on functional connectivity within or between brain networks [11–14]. However, most of these studies have focused on functional connectivity at only two time points (pre and posttreatment), thereby overlooking the dynamic temporal effects of antidepressant treatment, particularly during the early stage of therapy.

Previous studies have demonstrated that the effects of antidepressants on task‐evoked brain activation can emerge within days, preceding noticeable clinical symptom improvements [15, 16]. By adopting a longitudinal multitime‐point design that incorporates early treatment‐related changes in brain activity, researchers can better elucidate the evolving trajectories of brain function overtime. Such an approach holds promise for shedding new light on the neural mechanisms underlying interindividual variability in antidepressant efficacy [17]. Using this approach, several studies have shown that brain activity is dynamically affected by antidepressant treatment and that baseline brain activity or changes during treatment may be associated with or even predict treatment response [18–23]. Furthermore, differences in local spontaneous brain activity between remitters and nonremitters can be detected early in the course of treatment [18]. Divergent trajectories of functional connectivity within the executive control network and the default mode network between remitters and nonremitters have been observed during venlafaxine treatment for late‐life depression [23]. Despite these advances, the evolving trajectories of resting‐state functional connectivity (rsFC) throughout the treatment course in adults with MDD remain elusive.

The amygdala, a central structure involved in emotional regulation, sensory gating, and visceral control during affective processing [24, 25], is a key brain region whose dysfunction underlies the pathophysiology of MDD [26–31]. Previous studies have also indicated that the amygdala plays a significant role in responding to antidepressants. A meta‐analysis revealed that amygdala activity increased in response to positive emotional stimuli but decreased in response to negative emotional stimuli following repeated antidepressant treatment [32]. This finding has been supported by additional studies not included in that meta‐analysis [15, 33, 34]. Given the well‐established role of the 5‐hydroxytryptamine (5‐HT) system in modulating amygdala activity [35], these task‐state fMRI studies suggest that selective serotonin reuptake inhibitors (SSRIs) may alleviate depressive symptoms by normalizing amygdala hyperactivity through 5‐HT‐mediated pathways. For example, a study demonstrated a significant reduction in left amygdala activation in response to fearful and angry faces after 6 and 12 weeks of paroxetine treatment. Importantly, these neural changes were associated with clinical improvements, as reflected by reductions in 17‐item Hamilton Depression Rating Scale (HAMD) scores [36]. Several resting‐state fMRI studies have also reported alterations in amygdala rsFC in patients with MDD following antidepressant treatment. For instance, one study observed both increased and decreased rsFC between the amygdala and specific brain regions in adolescents with MDD after 8 weeks of SSRI treatment (fluoxetine or sertraline) [37]. Another study found that rsFC between the right amygdala and the lateral anterior cingulate cortex (ACC) was normalized after 12 weeks of SSRI treatment (escitalopram, fluoxetine, or sertraline), whereas rsFC between the left amygdala and the lateral ACC remained persistently impaired [38]. However, another study reported no significant changes in amygdala rsFC after 12 weeks of antidepressant treatment in adolescents with MDD [39]. In remitted adult patients with MDD, one study found decreased rsFC between the amygdala and the bilateral prefrontal cortex after 8 weeks of fluoxetine treatment [40].

Recent advances in amygdala research have revealed its intricate structural and functional heterogeneity, demonstrating that it comprises multiple subregions with distinct roles rather than functioning as a uniform neural nucleus [41–43]. Amunts [44] and colleagues divided the amygdala into three primary subregions, namely, the centromedial (CM), basolateral (BL), and superficial (SF) amygdala, based on the cytoarchitectonic features observed in the postmortem brain study. The CM amygdala, connected to the brainstem and striatum, is implicated in motor behavior and response preparation [45]; the BL amygdala, connected to the prefrontal cortex and thalamus, is involved in emotion and learning [41, 46]; and the SF amygdala, connected to multiple brain regions, is responsible for processing olfactory and emotional stimuli [25, 41]. Several fMRI studies have highlighted abnormal rsFC of the amygdala subregions in patients with MDD [28–31]. Two studies have explored the impact of antidepressant treatment on amygdala subregions. One study reported decreased rsFC between the left BL amygdala and the left precuneus in responders to ketamine infusion [47]. Another study found altered effective connectivity associated with specific amygdala subregions following SSRI treatment [48]. However, a comprehensive longitudinal study examining the differential trajectories of amygdala subregion rsFC in patients with distinct treatment outcomes remains lacking. Such research is critical for elucidating the mechanisms of antidepressant treatment, identifying biomarkers for predicting treatment response, optimizing clinical treatment strategies, and improving treatment outcomes.

Therefore, this study aimed to investigate how antidepressant treatment dynamically influences rsFC of amygdala subregions across different treatment response groups using an 8‐week longitudinal multitime point study (i.e., baseline, 2‐week posttreatment, and 8‐week posttreatment). In addition, this study explored the relationship between baseline rsFC and its longitudinal changes with baseline depressive symptoms and their improvements. Escitalopram, an SSRI that selectively targets the 5‐HT system and has demonstrated robust efficacy in comparative analyses of commonly prescribed antidepressants [49], was selected as the sole antidepressant to minimize treatment‐related variability. We hypothesized that (1) at baseline, remitters and nonremitters would exhibit abnormal rsFC of the amygdala subregions compared with healthy controls (HCs); (2) these rsFC patterns would show distinct trajectories during escitalopram treatment in the two MDD groups; and (3) baseline rsFC and their longitudinal changes would be significantly associated with depressive symptom severity and/or symptom improvement.

## 2. Materials and Methods

### 2.1. Participants and Antidepressant Treatment

Sixty patients with MDD were recruited from the outpatient service of Beijing Anding Hospital, Capital Medical University, via referral by a psychiatrist. All patients met the diagnostic criteria of DSM‐5 and were assessed using the Mini International Neuropsychiatric Interview (MINI), version 7.0.2 [50]. Inclusion criteria were as follows: age 18–65 years; first or recurrent depressive episode; a total score ≥ 14 on the HAMD; and no use of psychotropic medication at least 14 days prior to enrollment. MRI compatibility (i.e., the absence of metal or electronic implants) was confirmed. Exclusion criteria included a lifetime history of mania, hypomania, or bipolar disorder; any psychotic disorder; substance use disorder or acute intoxication; a score ≥3 on the HAMD suicide item; severe physical illness; and pregnancy or lactation. In parallel, 45 HCs were recruited through online advertisements. HCs were required to have no personal or family history of psychiatric disorders. The exclusion criteria for HCs were identical to those applied to patients with MDD.

Patients with MDD entered an 8‐week, open‐label treatment protocol with escitalopram. The initial dose was 5 mg/day and was adjusted based on periodic clinical evaluations, with a maximum dose of 20 mg/day. To alleviate insomnia, adjunctive sedative‐hypnotic medications (e.g., lorazepam, oxazepam, and zopiclone) were permitted when clinically indicated. Clinical assessments were conducted at baseline and at weeks 2, 4, 6, and 8, except that HAMD assessments were not performed at week 6. Resting‐state fMRI data were acquired at baseline and weeks 2 and 8. Patients were retrospectively classified into remission (MDDr) and nonremission (MDDnr) groups based on whether their total HAMD score was ≤7 at the end of the 8‐week period [51]. Analyses included patients who completed at least two fMRI scanning sessions and had available treatment outcome data at the end of the 8‐week follow‐up. HCs underwent clinical assessments and fMRI scanning only at baseline.

This study was approved by the Human Research and Ethics Committee of Beijing Anding Hospital, Capital Medical University. Written informed consent was obtained from all participants.

### 2.2. MRI Data Acquisition

MRI data were acquired using a 3.0 T Siemens Prisma scanner at the Department of Radiology, Beijing Anding Hospital, Capital Medical University. To reduce scanner noise and minimize head motion, participants were provided with earplugs and foam padding, respectively. For each participant, 200 volumes of resting‐state functional images and high‐resolution T1‐weighted structural images were acquired. During functional scanning, participants were instructed to remain awake, keep their eyes closed, stay relaxed, and not engage in any specific task. An echo‐planar imaging (EPI) sequence was used to acquire functional images with the following scan parameters: repetition time (TR)/echo time (TE) = 2000 ms/30 ms, flip angle (FA) = 90°, 33 axial interleaved slices, slice thickness/gap = 3.5 mm/0.7 mm, matrix size = 64 × 64, field of view (FOV) = 200 × 200 mm^2^, voxel size = 3.13 × 3.13 × 4.2 mm^3^, and total scan duration = 6 min 40 s. A magnetization‐prepared rapid gradient‐echo (MPRAGE) sequence was used for structural images with the following scan parameters: TR/TE = 2530 ms/1.85 ms, FA = 15°, 192 slices, slice thickness = 1 mm, sagittal scanning, matrix size = 256 × 256, FOV = 256 × 256 mm^2^, and voxel size = 1 × 1 × 1 mm^3^.

### 2.3. MRI Data Preprocessing

**Figure 1:**
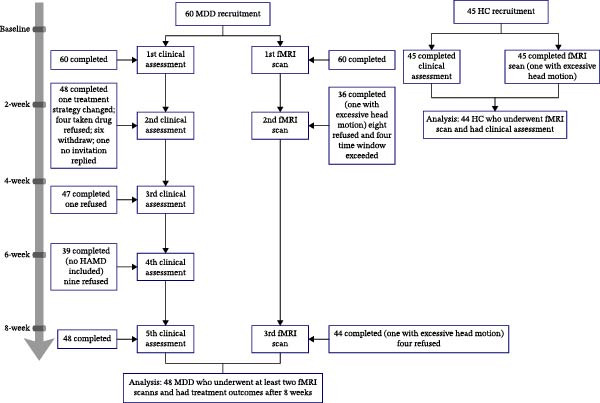
Study design and flowchart.

Brain images were processed using the Data Processing Assistant for Resting‐State fMRI (DPARSF version 5.3, http://rfmri.org/DPARSF), which is implemented in DPABI and based on SPM12 and MATLAB. The first five volumes of the functional images were discarded to allow for signal equilibration. The remaining images were corrected for slice timing and head motion. Nuisance covariates were regressed out, including the 24 Friston head‐motion parameters, the first five principal components of white matter and cerebrospinal fluid signals, and linear and quadratic trends. Functional images were spatially normalized to the Montreal Neurological Institute space using segmented T1 images and resampled to 2 mm isotropic voxels. The normalized images were then spatially smoothed using a 4 mm full‐width at half‐maximum Gaussian kernel and temporally band‐pass‐filtered (0.01–0.1 Hz). Head motion was quantified using volume‐based framewise displacement (FD) [52]. Participants with a mean FD exceeding three standard deviations from the sample mean or with more than 100 “bad” time points (FD > 0.5 mm) were excluded. Subsequently, scrubbing was performed by including “bad” time points as separate regressors in the model [53]. Ultimately, imaging data from one patient at the 2‐week time point, another at the 8‐week time point, and baseline data from one HC were excluded. Details of the longitudinal study design are illustrated in Figure 1.

### 2.4. Seed‐Based rsFC Analysis of the Amygdala Subregions

Detailed procedures for defining the amygdala subregions and calculating rsFC have been described in our previous studies [54, 55]. Briefly, the SPM Anatomy Toolbox was used to define the amygdala subregions, including the CM, BL, and SF nuclei, with the left and right hemispheres analyzed separately [44, 56]. For each subregion, seed‐based rsFC analysis was performed by computing Pearson’s correlation coefficients between the mean time series of the seed region and the time series of all other voxels across the whole brain. The resulting correlation coefficients were then transformed into z‐scores using Fisher’s *r*‐to‐*z* transformation to improve normality. These z‐score maps for each subregion were subsequently entered into group‐level analyses.

As a supplementary analysis, the rsFC of the whole amygdala was also examined. The whole amygdala (left and right hemispheres analyzed separately) was defined by combining the masks of three subregions within each hemisphere, and the same seed‐based rsFC analysis pipeline as used for the subregion analyses was applied.

### 2.5. Statistical Analysis

Demographic and clinical characteristics were compared among the MDDr, MDDnr, and HC groups using Kruskal–Wallis tests for continuous variables (including nonnormally distributed variables such as age and “bad” time points) and chi‐square tests for categorical variables. For between‐group comparisons of clinical assessment scores (MDDr vs. MDDnr), independent‐samples *t*‐tests were applied to normally distributed data, whereas Mann–Whitney *U* tests were used for nonnormally distributed variables. All analyses were performed using SPSS version 26.0.

For rsFC analyses, baseline differences in amygdala subregion connectivity among the MDDr, MDDnr, and HC groups were examined using a general linear model in SPM 12, with age, gender, education level, and mean FD included as covariates. Multiple comparisons were corrected using cluster‐level family‐wise error (FWE) correction in SPM12. A cluster‐defining threshold of *p* < 0.001 (uncorrected) was first applied to identify spatially contiguous voxels. Cluster‐level significance was then determined based on Gaussian random field theory, incorporating spatial smoothness estimation (derived from model residuals following application of a 4 mm full width at half‐maximum Gaussian kernel during preprocessing) and topological inference (accounting for the expected number of resels in the search volume). Only clusters surviving both the voxel‐wise threshold and cluster‐level FWE correction were considered statistically significant. To further control for multiple comparisons across amygdala subregions, Bonferroni correction was applied, with statistical significance set at *p* < 0.008 (corrected for six subregion comparisons). For completeness, results that did not survive Bonferroni correction were also reported as exploratory findings given the novelty of subregion‐specific analyses. Subsequent analyses focused on these identified rsFCs regardless of Bonferroni correction. In particular, rsFCs showing differential patterns between MDDr and MDDnr groups relative to HCs at baseline were considered potential predictors of treatment outcomes. Post hoc analyses were conducted by extracting the mean connectivity strength of each significant cluster. False‐discovery rate (FDR) correction was applied in post hoc analyses, with statistical significance set at corrected *p* < 0.05.

Longitudinal changes in rsFC were examined using a linear mixed‐effects model implemented with the *lmer* function in R version 4.2.1. Group (MDDr vs. MDDnr), time (baseline, 2‐week, and 8‐week posttreatment), and their interaction were included as fixed effects, with subject‐specific intercepts modeled as random effects. Age, gender, education level, and mean FD were included as nuisance variables [57]. Details of this modeling approach have been described in our previous study [11]. When a significant group × time interaction was observed, simple effects analyses were performed. Multiple comparisons across time points were controlled using FDR correction, with statistical significance set at corrected *p* < 0.05. A significant main effect of group was interpreted as a time‐invariant effect, whereas a significant main effect of time indicated a time‐dependent effect. In the latter case, the latent growth curve model (LGCM) was further applied to characterize the relationship between longitudinal rsFC changes and clinical symptoms. In addition, to evaluate normalization effects, separate linear regression analyses, controlling for age, gender, education level, and mean FD, were conducted to compare rsFC values in the MDDr and MDDnr groups with those in HCs (available only at baseline) at the 2‐ and 8‐week time points. Finally, partial correlation analyses were performed to examine the clinical correlates of rsFC and its longitudinal changes. Age, gender, education level, and mean FD (or changes in FD) were included as covariates. Specifically, correlations were assessed between (1) baseline rsFC and baseline HAMD scores; (2) baseline rsFC and HAMD score reductions at 2‐, 4‐, or 8‐week; (3) rsFC changes at 2‐week and HAMD score reductions at 2‐, 4‐, or 8‐week; (4) rsFC changes at 8‐week and HAMD score reductions at 8‐week. All correlation analyses were two‐tailed. Bonferroni correction was applied to adjust for multiple comparisons, with statistical significance set at *p* < 0.006 (corrected for eight correlation analyses). Uncorrected results were also reported to aid interpretation in this exploratory analysis. The same analytical procedures were applied to the whole amygdala connectivity (left and right amygdala hemispheres analyzed separately).

### 2.6. Sensitivity Analyses

To mitigate potential bias arising from unbalanced missing fMRI data across longitudinal time points (i.e., different subsets of participants missing scans at 2‐ vs. 8‐week follow‐up), we conducted a sensitivity analysis restricted to patients with complete data at all three time points (15 MDDr and 15 MDDnr), thereby enabling more robust comparisons of longitudinal rsFC trajectories. In addition, given evidence suggesting that lorazepam may exert confounding effects on fMRI signals, particularly within the amygdala [58, 59], we further stratified the sample into patients receiving lorazepam (7 MDDr and 11 MDDnr) and those not receiving lorazepam (18 MDDr and 12 MDDnr). RsFC trajectories were then analyzed separately within each subgroup.

## 3. Results

### 3.1. Demographic and Clinical Variables

Forty‐eight patients with MDD completed at least two fMRI scanning sessions and had evaluable treatment outcomes at the 8‐week time point. Of these, 48 completed baseline fMRI data (25 MDDr and 23 MDDnr), 35 completed a 2‐week scan (20 MDDr and 15 MDDnr), and 43 completed a 8‐week scan (20 MDDr and 23 MDDnr). These data were included in subsequent analyses. In addition, 44 HCs were included. Detailed demographic and clinical characteristics are presented in Table 1.

**Table 1:** Demographic and clinical information.

Characteristics	MDDr (*N* = 25)	MDDnr (*N* = 23)	HC (*N* = 44)	*H/t/Z/χ* ^2^	*p*
Age (median, IQR)	28 (25.5–35.5)	25 (24–31)	26 (24–28)	3.845	0.146^d^
Gender (M/F)	6/19	6/17	16/28	1.425	0.490^g^
Educational level	9/9/7	2/14/7	7/16/21	8.747	0.064^g^
Episode type (*f*/*r*)	12/13	6/17	—	2.454	0.117^g^
Mean FD^a^ (median, IQR)	0.137 (0.099–0.198)	0.167 (0.101–0.227)	0.128 (0.099–0.180)	1.208	0.547^d^
“Bad” time points^a^ (median, IQR)	1 (0–4)	3 (0–11)	1 (0–5.75)	2.209	0.331^d^
HAMD‐baseline	21.28 ± 3.43	21.04 ± 3.50	—	0.236	0.814^e^
HAMD‐2 w	11.16 ± 4.71	16.26 ± 4.29	—	−3.910	<0.001 ^∗^^e^
HAMD‐4 w	8.00 ± 3.98	14.09 ± 4.71	—	−4.806	<0.001 ^∗^^e^
HAMD‐8 w	3.68 ± 2.25	13.04 ± 4.50	—	−9.003	<0.001 ^∗^^e^
Escitalopram dosage (mg/d)^b^ (median, IQR)	10 (10–10)	10 (10–10)	—	288.500	0.952^f^
Escitalopram dosage (mg/d)^c^ (median, IQR)	15 (10–15)	15 (15–20)	—	370.000	0.065^f^
Sedative‐hypnotic (Y/N)^b^	11/14	16/7	—	3.181	0.074^g^
Sedative‐hypnotic (Y/N)^c^	7/18	11/12	—	2.009	0.156^g^

*Note:* M: male; F: female. f: first‐episode; r: recurrent‐episode. Y: yes; N: no. IQR: interquartile range. Educational level was divided into three categories: junior‐senior high school, junior college/undergraduate/postgraduate, or above. Group comparisons were performed with the following test: Kruskal–Wallis test (d), two‐sample *t*‐test (e), Mann–Whitney *U* test (f), and Chi‐square test (g), as appropriate for the data distribution and variable type. Significant differences were marked by asterisks ( ^∗^).

^a^Baseline.

^b^2‐week of follow‐up.

^c^8‐week of follow‐up.

At baseline, there were no significant differences among the MDDr, MDDnr, and HC groups in age, gender, education level, mean FD, and the number of “bad” time points (*H* = 3.845, *p* = 0.146; *χ* ^2^ = 1.425, *p* = 0.490; *χ* ^2^ = 8.747, *p* = 0.064; *H* = 1.208, *p* = 0.547; *H* = 2.209, *p* = 0.331). There were also no significant differences between the MDDr and MDDnr groups in episode type or baseline HAMD scores (*χ* ^2^ = 2.454, *p* = 0.117; *t* = 0.236, *p* = 0.814). At follow‐up, HAMD scores were significantly lower in the MDDr group than in the MDDnr group at 2‐week, 4‐week, and 8‐week time points (*t* = −3.910, *p* < 0.001; *t* = −4.806, *p* < 0.001; *t* = −9.003, *p* < 0.001). The two groups were comparable in escitalopram dosage and the proportion of sedative‐hypnotic use at both the 2‐week and 8‐week time points (*Z* = 288.500, *p* = 0.952; *Z* = 370.000, *p* = 0.065; *χ* ^2^ = 3.181, *p* = 0.074; *χ* ^2^ = 2.009, *p* = 0.156).

### 3.2. Group Differences in rsFC of the Amygdala Subregions at Baseline

**Figure 2:**
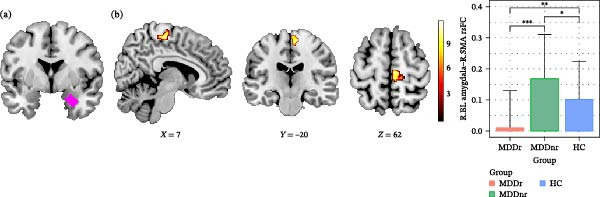
Differences in baseline rsFC among the MDDr, MDDnr, and HC groups. (a) Seed region located in the right BL amygdala. (b) Group differences in baseline rsFC between the right BL amygdala and the right SMA.  ^∗^ *p* < 0.05,  ^∗∗^ *p* < 0.01,  ^∗∗∗^ *p* < 0.001. BL amygdala, basolateral amygdala; SMA, supplementary motor area.

After Bonferroni correction across seed regions (adjusted significance threshold *p*_FWE_ < 0.008), no significant group differences in amygdala subregion rsFC were observed (all *p*_FWE_ > 0.008). Exploratory analyses revealed a group difference in rsFC between the right BL amygdala and a cluster encompassing the right supplementary motor area (SMA) (*p*_FWE_ = 0.041; Figure 2a,b, and Table S1). Post hoc comparisons showed that this rsFC was significantly lower in MDDr group than in both the HC and MDDnr groups (*t* = −3.462, *p*_FDR_ = 0.001; *t* = −5.112, *p*_FDR_ < 0.001, respectively), whereas the MDDnr group exhibited higher rsFC than HCs (*t* = 2.343, *p*_FDR_ = 0.022).

### 3.3. Effects of Antidepressant Treatment on the Identified rsFC of the Right BL Amygdala

**Figure 3:**
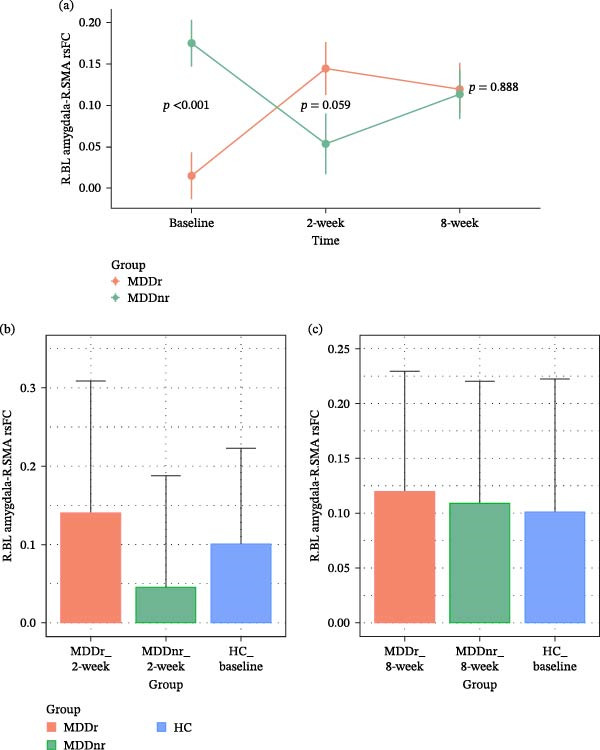
Longitudinal analysis of the identified rsFC in the MDDr and MDDnr groups: (a) Significant group × time interaction effect in rsFC between the right BL amygdala and the right SMA. (b) Comparisons of rsFC between the right BL amygdala and the right SMA at 2‐week time point between patients (MDDr or MDDnr) and HCs (available only at baseline). (c) Comparisons of rsFC between the right BL amygdala and the right SMA at 8‐week time point between patients (MDDr or MDDnr) and HCs (available only at baseline). BL amygdala, basolateral amygdala; SMA, supplementary motor area.

A significant group × time interaction was observed in rsFC between the right BL amygdala and the right SMA (*F* = 9.939, *p* < 0.001, ηp^2^ = 0.19; Figure 3a and Table 2), indicating distinct longitudinal trajectories in the MDDr and MDDnr groups. Simple effects analyses showed that in the MDDr group, rsFC increased from baseline to 2‐ or 8‐week time points (*t* = −3.275, *p*_FDR_ = 0.005, Cohen’s *d* = −0.994; *t* = −2.647, *p*_FDR_ = 0.015, Cohen’s *d* = −0.803), with no significant change between 2‐ and 8‐week time point (*t* = 0.593, *p*_FDR_ = 0.555, Cohen’s *d* = 0.190). In contrast, in the MDDnr group, rsFC decreased from baseline to 2‐week time points (*t* = 2.779, *p*_FDR_ = 0.020, Cohen’s *d* = 0.933), with no significant changes from baseline to 8‐week time point or between the 2‐ and 8‐week time points (*t* = 1.595, *p*_FDR_ = 0.173, Cohen’s *d* = 0.471; t = −1.374, *p*_FDR_ = 0.173, Cohen’s *d* = −0.462). At baseline, rsFC was lower in the MDDr group than in the MDDnr group (*t* = −3.981, *p* < 0.001, Cohen’s *d* = −1.228), whereas no group differences were observed at 2‐ or 8‐week time points (*t* = 1.910, *p* = 0.059, Cohen’s *d* = 0.698; *t* = 0.141, *p* = 0.888, Cohen’s *d* = 0.046).

**Table 2:** The interaction effect, group effect, and time effect in rsFC of the right BL amygdala.

RsFC	MDDr_0	MDDr_2	MDDr_8	MDDnr_0	MDDnr_2	MDDnr_8	Interaction *F*/*p*/ηp^2^	Group *F*/*p*/ηp^2^	Time *F*/*p*/ηp^2^
Right BL amygdala‐R.SMA	0.01 ± 0.03	0.14 ± 0.03	0.12 ± 0.03	0.18 ± 0.03	0.05 ± 0.04	0.11 ± 0.03	9.939/ < 0.001/0.19	0.574/0.453/0.01	0.334/0.717/0.008

*Note:* The functional connectivity values of different groups at different time points were represented by estimated marginal means ± standard error. 0: baseline; 2: 2‐week of follow‐up; 8: 8‐week of follow‐up.

Abbreviations: BL amygdala, basolateral amygdala; SMA, supplementary motor area.

No significant main effect of group or time was observed in this rsFC (*F* = 0.574, *p* = 0.453, ηp^2^ = 0.01; *F* = 0.334, *p* = 0.717, ηp^2^ = 0.008). Consequently, no further LGCM analysis was conducted.

### 3.4. Comparisons of the Identified rsFC Between Each Patient Group (MDDr and MDDnr) and HCs Following Antidepressant Treatment

To assess normalization, planned comparisons were conducted to compare rsFC values in each patient group (MDDr and MDDnr) with those in HCs (available only at baseline) at 2‐ and 8‐week time points.

Regarding the rsFC between the right BL amygdala and the right SMA, no significant differences were observed between either patient group and HCs at 2‐week (*b* = 0.014, *p* = 0.728; *b* = −0.050, *p* = 0.227; Figure 3b and Table S2) or 8‐week time points (*b* = 0.000, *p* = 0.990; *b* = 0.004, *p* = 0.895; Figure 3c and Table S2).

### 3.5. Clinical Correlates of the Identified rsFC and Their Changes

**Figure 4:**
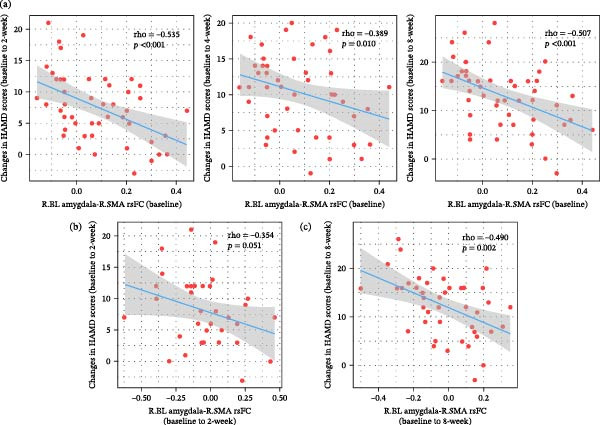
Clinical correlates of the identified rsFC and their longitudinal changes. (a) Clinical correlates of the rsFC between the right BL amygdala and the right SMA at baseline. (b) Clinical correlates of the rsFC between the right BL amygdala and the right SMA from baseline to 2‐week time point. (c) Clinical correlate of the rsFC between the right BL amygdala and the right SMA from baseline to 8‐week time point. BL amygdala, basolateral amygdala; SMA, supplementary motor area.

After Bonferroni correction for multiple correlations (adjusted significance threshold *p* < 0.006), baseline rsFC between the right BL amygdala and the right SMA were significantly negatively correlated with HAMD score reductions at both 2‐ and 8‐week time points (rho = −0.535, *p* < 0.001; rho = −0.507, *p* < 0.001). A similar association was observed at the 4‐week time point at an uncorrected level (rho = −0.389, *p* = 0.010). These findings suggested that lower baseline rsFC were more likely to experience early therapeutic benefits (2 weeks) and sustained efficacy through 8 weeks. Changes in rsFC from baseline to the 8‐week time point were negatively correlated with HAMD score reductions at 8‐week time point after correction (rho = −0.490, *p* = 0.002), with a marginal trend at the 2‐week time point (rho = −0.354, *p* = 0.051), suggesting that changes in rsFC coincided with improvement of clinical symptoms. No significant correlations were observed between rsFC changes at the 2‐week time point and HAMD score reductions at the 4‐ or 8‐week time points (rho = −0.256, *p* = 0.173; rho = −0.272, *p* = 0.139), nor between baseline rsFC and baseline HAMD scores (rho = −0.207, *p* = 0.177) (Figure 4a–c).

### 3.6. Results of the Whole Amygdala

Results of the whole amygdala connectivity analyses are provided in the Supporting Information.

### 3.7. Sensitivity Analyses

Sensitivity analyses supported the robustness of the findings. Analyses restricted to participants with complete longitudinal data yielded results consistent with the primary analysis, demonstrating distinct rsFC trajectories between the MDDr and MDDnr groups (Figure S4 and Table S6). Stratified analyses by lorazepam use showed that these differential trajectories were present only in patients not receiving lorazepam, replicating the primary findings (Figure S5 and Table S7), whereas no such effects were observed among lorazepam users. Detailed results are provided in the Supporting Information.

## 4. Discussion

The present study identified distinct trajectories of rsFC in the right BL amygdala between the MDDr and MDDnr groups using a longitudinal multitime point design. At baseline, rsFC between the right BL amygdala and the right SMA was lower in the MDDr group but higher in the MDDnr group compared with HCs, although this difference did not survive stringent multiple comparisons correction. Importantly, the trajectories of rsFC during treatment differed between the two patient groups. Notably, this rsFC normalized after 2 weeks of treatment and persisted through 8 weeks. Sensitivity analysis yielded consistent results, supporting the robustness of these findings. Moreover, correlation analyses demonstrated that baseline rsFC was associated with subsequent treatment response and that rsFC changes were aligned with symptom improvement, although some correlations did not survive stringent multiple comparisons correction. Taken together, these findings should be interpreted with caution but provide preliminary insights into the neural mechanisms underlying differential antidepressant responses and highlight the importance of considering subregional amygdala connectivity in treatment stratification.

At baseline, the right BL amygdala showed differential rsFC with the right SMA among patients with MDDr, MDDnr, and the HCs, although this contrast did not survive multiple comparisons correction. Previous studies have suggested that the amygdala and the prefrontal cortex, including the SMA, share extensive and reciprocal connections. Through these pathways, the amygdala can exert bottom‐up modulation on prefrontal activity, while the prefrontal cortex exerts top‐down control over amygdala‐mediated responses to emotionally salient stimuli [60, 61]. It is noteworthy that our study primarily involved the right amygdala. The right amygdala has been reported to show greater sensitivity to pictorial or image‐related affective information and to play a more prominent role in the rapid detection and processing of emotional stimuli compared with the left amygdala [62]. As part of a dynamic emotional stimulus detection system, the right amygdala is also involved in monitoring fluctuations in dynamic emotions and facilitating habituation to repeated negative stimuli. Disruption of this habituation process may increase vulnerability to persistent negative affect and rumination in depression [63]. Among amygdala subregions, the BL amygdala plays a significant role in emotional processing and learning due to its strong connections with the prefrontal cortex and thalamus [41, 46]. Beyond this local circuitry, the observed differential rsFC between the right BL amygdala and the right SMA may reflect dysfunction within a broader cortico‐subcortical network implicated in MDD. The SMA is increasingly recognized not only as a motor‐related region but also as a key hub integrating emotional, cognitive, and motor processes. For example, altered functional connectivity between the SMA and posterior cingulate cortex has been associated with psychomotor performance deficits in mood disorders [64]. In addition, disrupted structure‐function coupling of the SMA has been associated with cognitive impairments in MDD, suggesting that SMA abnormalities may reflect system‐level network dysfunction rather than isolated regional changes [65]. Functional alterations in the SMA have also been observed in MDD patients with prominent somatic symptoms, further supporting its role in integrating emotional and bodily states [66]. Moreover, task‐based study has demonstrated abnormal SMA connectivity during motor execution, highlighting its relevance to psychomotor disturbances, a core but often under‐characterized feature of depression [67]. Although psychomotor performance was not directly assessed in the present study, the differential rsFC between the right BL amygdala and the right SMA observed here may reflect disruptions in emotion‐motor integration circuits. Such disruptions may contribute to clinical features, including reduced motivation, psychomotor retardation, or agitation. Future studies incorporating behavioral measures and longitudinal assessments of psychomotor function are warranted to further elucidate the functional significance of this circuit. Consistent with this broader network perspective, several studies have reported altered rsFC between the amygdala and SMA across psychiatric conditions. For instance, adolescents engaging in nonsuicidal self‐injury exhibited increased rsFC between the amygdala and the SMA compared with controls [68]. Patients with comorbid posttraumatic stress disorder and MDD showed divergent connectivity patterns between amygdala subregions and the SMA [69], while hemodialysis patients with depressed mood also demonstrated increased rsFC between the amygdala and the SMA [70]. Together, these findings suggest that the amygdala‐SMA circuit may represent a transdiagnostic pathway involved in affective dysregulation.

Notably, baseline rsFC between the right BL amygdala and the right SMA exhibited distinct abnormal patterns in the MDDr and MDDnr groups relative to HCs. Moreover, this rsFC showed correlations with both early treatment response and response at 4 weeks and 8 weeks, although some correlations did not survive after multiple comparisons correction. The finding suggested that this rsFC may reflect the neural basis of interindividual variability in treatment response and may serve as a potential prognostic biomarker in MDD. Relatively few studies have examined the relationship between the amygdala function and interindividual differences in treatment response. In a task‐state fMRI study, responders to 8‐week antidepressant treatment (escitalopram, sertraline, and venlafaxine‐XR) showed decreased amygdala reactivity to subliminal threat or happy facial expression at baseline compared with HCs, whereas nonresponders did not differ significantly from HCs, except for venlafaxine‐XR nonresponders, who exhibited hyperreactivity to subliminal sadness at baseline [71]. Another study reported baseline rsFCs between the amygdala and the lateral and medial prefrontal cortices were increased in a cohort of patients with MDD (*n* = 40), with a majority (*n* = 25) achieving remission after 8 weeks of fluoxetine treatment [40]. Additional evidence linking amygdala functional activity to antidepressant efficacy emerges from ketamine studies. For example, baseline rsFC between the amygdala and the right subgenual anterior cingulate gyrus differed significantly between eventual responders and nonresponders in treatment‐resistant depression [72]. Similarly, in anxious depression, baseline rsFC between the left BL amygdala and left precuneus was higher in responders than in nonresponders [47], whereas heightened pretreatment rsFC between the amygdala and the left SMA and the right precentral gyrus forecasted poorer 8‐week SSRI outcomes [73], consistent with our findings. Taken together, these findings, along with our own, highlight baseline functional activity and connectivity of the BL amygdala subregion as a promising neural signature for discriminating antidepressant efficacy and forecasting individual treatment response. Admittedly, not all baseline or correlation results withstood stringent multiple comparisons correction, a limitation plausibly attributable to the small sample size. Nevertheless, given the exploratory nature of this study and the novelty of the amygdala subregion‐specific, longitudinal modeling, the uncorrected trends retain meaningful heuristic value for guiding future large‐scale validation efforts.

In the longitudinal analysis, rsFC between the right BL amygdala and the right SMA exhibited distinct trajectories in MDDr and MDDnr groups during escitalopram treatment, and these findings were further substantiated in a sensitivity analysis. To our knowledge, no prior longitudinal multitime point fMRI study has charted how amygdala connectivity evolves differentially across divergent antidepressant outcomes. Earlier two‐time point studies have reported different patterns of change in amygdala functional activity between responders and nonresponders following antidepressant treatment. For instance, one resting‐state fMRI study identified significant differences in rsFC changes involving the bilateral CM amygdala and the left orbital part of the superior frontal gyrus, as well as the left BL amygdala and the right middle frontal gyrus, following six intravenous ketamine infusions over a 2‐week period [74]. Another task‐state fMRI study found that responders exhibited increased amygdala reactivity to subliminal threat or happy facial expressions, with this increase trending towards normalization, whereas nonresponders showed decreased or downward‐trending reactivity after 8 weeks of antidepressant treatment [71]. These previous findings, along with our own, suggest that antidepressant treatment may exert differential treatment efficacy through its impact on amygdala function across individuals. By contrast, a recent deep brain stimulation trial reported increased connectivity between the right amygdala and the sensorimotor cortices underactive stimulation compared with sham; however, this change did not discriminate responders from nonresponders, possibly due to limited statistical power associated with a small sample size [75].

In the current study, we observed that rsFC between the right BL amygdala and the right SMA increased from baseline to 2‐week time point in the MDDr group, whereas it decreased in the MDDnr group. Several studies have explored the early effects of antidepressants on the amygdala. For example, one study reported that a 7‐day antidepressant intervention reduced rsFC between the amygdala and the ventral medial prefrontal and orbitofrontal cortex in healthy volunteers compared with the placebo group [76]. In another study, ketamine treatment was associated with increased rsFC between the left amygdala and the left medial superior frontal gyrus following six intravenous infusions over a 2‐week period, and this change correlated with symptom improvement [77]. Previous studies have suggested that early changes in rsFC following antidepressant treatment may be related to tolerability issues, such as “activation”, which includes symptoms like anxiety, impulsivity, insomnia, and restlessness that manifest early in treatment as a side effect of SSRIs and are associated with SSRI plasma concentration. These effects may be tied to the impact of SSRIs on the amygdala‐prefrontal circuit [78–81]. The opposite direction of change observed at 2‐week may reflect differential early neural adaptation. Specifically, increased rsFC in the MDDr group may indicate a rapid restoration of functional integration between emotional processing and motor preparation systems, whereas decreased connectivity in the MDDnr group may reflect persistent or even exacerbated dysregulation within this circuit. Interestingly, the two groups appeared to converge by 8‐week, suggesting that these early differences may be transient and state‐dependent rather than trait‐like. One possible explanation may be that early‐phase neural plasticity differentiates treatment responders from nonresponders, whereas later‐stage changes may reflect delayed treatment effects, compensatory processes, or reduced interindividual variability overtime. This dynamic pattern highlights the importance of considering temporal trajectories of functional connectivity rather than relying solely on endpoint comparisons. These findings may have implications, such as early changes in rsFC between the amygdala and the SMA could serve as a potential biomarker for predicting treatment response, and this circuit may represent a promising target for neuromodulation interventions aimed at improving emotional‐motor integration. Taken together, the early divergence and later convergence of rsFC between the amygdala and the SMA connectivity may reflect a dynamic process of neural reorganization, in which successful treatment is characterized by rapid restoration of limbic‐motor integration, whereas persistent dysregulation may underlie nonresponse.

This study has several limitations. First, the relatively small sample size may have reduced statistical power and contributed to the loss of significance in some findings after multiple comparisons correction across amygdala subregions or correlation analyses. In addition, missing fMRI data, particularly at the 2‐week follow‐up, may have introduced bias or further reduced statistical power in the longitudinal analyses. Nevertheless, the primary findings remained robust and were supported by sensitivity analyses. Second, the limited sample size constrained our ability to perform a comprehensive whole‐brain longitudinal analysis of the rsFC involving the amygdala and its subregions, as such analyses would require multiple comparison corrections and might obscure meaningful effects. Therefore, we restricted longitudinal analysis to connections that showed group differences at baseline. This approach may have overlooked other relevant connections, and the absence of differences at baseline does not necessarily imply insensitivity to antidepressant treatment. This limitation may also partly explain the lack of observed time‐varying rsFC changes in some amygdala circuits. Future studies should extend this work by examining broader amygdala‐related networks informed by prior evidence. Third, only baseline fMRI data were collected for HCs in this study. The absence of longitudinal data in this group limits our ability to account for test‐retest effects and to characterize the natural temporal variability of the brain function. Future studies should include longitudinal assessments in HCs to address this issue. Fourth, the spatial resolution of the current imaging protocol may have limited the precise characterization of amygdala subregions. A previous 7T ultrahigh‐field MRI study reported no significant volumetric differences in amygdala subregions between MDD and HC groups [82]. Future studies using ultrahigh‐field MRI may provide more fine‐grained validation of the present findings. Fifth, the relatively short resting‐state acquisition duration (6 min 40 s) may limit the reliability of functional connectivity estimates as longer scan durations are generally recommended for robust resting‐state fMRI measurements [83]. Although rigorous preprocessing and quality control procedures were applied, future studies with longer acquisition times would likely improve the stability and reliability of connectivity estimates. Finally, although stratification by lorazepam use supported the robustness of our main findings in the nonusers, the absence of significant rsFC trajectory differences in lorazepam users suggests that benzodiazepines may obscure functional connectivity patterns relevant to antidepressant response. Future studies with larger samples should explicitly model the potential confounding effects of benzodiazepines and other sedative medications.

## 5. Conclusion

In summary, our longitudinal multitime point design revealed distinct trajectories of rsFC in the right BL amygdala between the MDDr and MDDnr groups during an 8‐week treatment with escitalopram. These findings highlight the importance of capturing early treatment‐induced changes in rsFC as potential indicators of antidepressant response. Our results provide novel insights into the neural mechanisms underlying antidepressant efficacy and help to elucidate the basis of interindividual variability in treatment outcomes. Ultimately, this work may inform the development of predictive biomarkers and targeted interventions, thereby contributing to the advancement of personalized and precision treatment strategies for depression.

## Author Contributions

Gang Wang, Yuan Zhou, and Zhifang Zhang conceived and designed the study. Shudong Zhang performed the analysis and drafted the manuscript. Shudong Zhang, Zhifang Zhang, Xiaoya Li, Jingjing Zhou, Ruinan Li, Rui Liu, Yun Wang, Xiongying Chen, Yuan Feng, and Ling Zhang conducted the data collection and collation. Jian Cui provided some methodological support. Gang Wang and Yuan Zhou revised the manuscript critically.

## Funding

The study was supported by the Brain Science and Brain‐like Intelligence Technology‐National Science and Technology Major Project (Grant 2021ZD0200600), the National Natural Science Foundation of China (Grant 82071531), and the Beijing Hospitals Authority Youth Programme (Grant QML20211901).

## Conflicts of Interest

The authors declare no conflicts of interest.

## Supporting Information

Additional supporting information can be found online in the Supporting Information section.

## Supporting information


**Supporting Information** To improve the readability and conciseness of the main text, additional analyses and detailed results are presented in the supporting information. The difference in rsFC between the right BL amygdala and the right SMA among the MDDr, MDDnr, and HC groups at baseline is summarized in Table S1. Detailed information on the identified rsFC normalization effects following antidepressant treatment is provided in Table S2. Furthermore, supplementary analyses of the whole amygdala connectivity were conducted. Detailed results regarding baseline group differences, longitudinal changes in the identified rsFC, normalization effects following antidepressant treatment, and their clinical correlates are presented in Figures S1–S3 and Tables S3–S5. In addition, sensitivity analyses using complete longitudinal data, as well as stratification analyses based on lorazepam use, were performed. Detailed findings from these analyses are provided in Figures S4–S5 and Tables S6–S7.

## Data Availability

Data are available upon request from the corresponding author.
