# Supplementary material for: Distinct Trajectories of Amygdala Connectivity Patterns Characterize Remission vs. Non‐Remission in Patients With Major Depressive Disorder
Source: Depress Anxiety. 2026 Jun 11;2026:4701907. doi: 10.1155/da/4701907 (PMC13254815; doi:10.1155/da/4701907)
Supplement: Supplementary file 1 — Supporting Information To improve the readability and conciseness of the main text, additional analyses and detailed results are presented in the supporting information. The difference in rsFC between the right BL amygdala and the right SMA among the MDDr, MDDnr, and HC groups at baseline is summarized in Table S1. Detailed information on the identified rsFC normalization effects following antidepressant treatment is provided in Table S2. Furthermore, supplementary analyses of the whole amygdala connectivity were conducted. Detailed results regarding baseline group differences, longitudinal changes in the identified rsFC, normalization effects following antidepressant treatment, and their clinical correlates are presented in Figures S1–S3 and Tables S3–S5. In addition, sensitivity analyses using complete longitudinal data, as well as stratification analyses based on lorazepam use, were performed. Detailed findings from these analyses are provided in Figures S4–S5 and Tables S6–S7. [file DA-2026-4701907-s001.docx]

**Supplemental Materials**

Distinct Trajectories of Amygdala Connectivity Patterns Characterize Remission vs. Non-Remission in Patients with Major Depressive Disorder

**Contents**

**Section 1 Supplementary analyses on the rsFC of the whole amygdala**

**Section 2 Sensitivity analyses**

**Section 1 Supplementary analyses on the rsFC of the whole amygdala**

**Group differences in rsFC of the whole amygdala at baseline**

After Bonferroni correction across seed regions (adjusted significance threshold *p*_FWE_ < 0.025), a significant group difference was observed in rsFC between the right amygdala and a cluster encompassing the left supplementary motor area (SMA)/medial superior frontal gyrus (mSFG) among the MDDr, MDDnr, and HC groups (*p*_FWE_ = 0.001; Figure S1a, b, and Table S3). Post-hoc comparison showed that this rsFC was significantly lower in MDDr group than in both the HC and MDDnr groups (t = -3.407, *p*_FDR_ = 0.002; t = -5.372, *p*_FDR_ < 0.001, respectively), whereas the MDDnr group exhibited higher rsFC than HCs (t = 2.692, *p*_FDR_ = 0.009).

No significant group differences were observed in rsFC of the left amygdala.

**Effects of antidepressant treatment on the identified rsFC of the right amygdala**

A significant group × time interaction was observed in rsFC between the right amygdala and the left SMA/mSFG (F = 4.559, *p* = 0.013, ηp² = 0.10; Figure S2a and Table S4), indicating distinct longitudinal trajectories in the MDDr and MDDnr groups. Simple effects analyses showed that in the MDDr group, rsFC increased from baseline to 2-week time point (t = -2.465, *p*_FDR_ = 0.048, Cohen’s d = -0.748), with no significant changes from baseline to 8-week time point or between the 2- and 8-week time points (t = -0.817, *p*_FDR_ = 0.416, Cohen’s d = -0.248; t = 1.558, *p*_FDR_ = 0.184, Cohen’s d = 0.500). In contrast, in the MDDnr group, no significant changes in rsFC were observed across any time intervals (baseline to 2-week time point: t = 1.812, *p*_FDR_ = 0.220, Cohen’s d = 0.609; baseline to 8-week time point: t = 1.394, *p*_FDR_ = 0.251, Cohen’s d = 0.412; 2- to 8-week time points: t = -0.586, *p*_FDR_ = 0.559, Cohen’s d = -0.197). At baseline and 8-week time point, rsFC were significantly lower in the MDDr group than in the MDDnr group (t = -4.993, *p* < 0.001, Cohen’s d = -1.551; t = -2.698, *p* = 0.008, Cohen’s d = -0.891), whereas no group difference was observed at 2-week time point (t = -0.527, *p* = 0.599, Cohen’s d = -0.194).

No significant main effect of time was observed in this rsFC (F = 0.218, *p* = 0.805, ηp² = 0.005). Consequently, no further LGCM analysis was conducted.

**Comparisons of the identified rsFC between each patient group (MDDr and MDDnr) and HCs following antidepressant treatment**

Regarding the rsFC between the right amygdala and the left SMA/mSFG, no significant differences were observed between either patient group and HCs at 2-week (b = -0.038, *p* = 0.299; b = 0.024, *p*= 0.551; Figure S2b and Table S5) or 8-week time points (b = -0.067, *p* = 0.080; b = 0.046, *p* = 0.163; Figure S2c and Table S5).

**Clinical correlates of the identified rsFC and their changes**

After Bonferroni correction for multiple correlations (adjusted significance threshold *p* < 0.006), baseline rsFC between the right amygdala and the left SMA/mSFG were significantly negatively correlated with HAMD score reductions at both 4- and 8-week time points (rho = -0.501, *p* = 0.001; rho = -0.490, *p* = 0.001). A similar association was observed at the 2-week time point at an uncorrected level (rho = -0.372, *p* = 0.013). These findings suggested that lower baseline rsFC were more likely to experience early therapeutic benefits (2 weeks) and sustained efficacy through 8 weeks. Changes in rsFC from baseline to the 2-week time point were negatively correlated with HAMD score reductions at 4-week time point after correction (rho = -0.522, *p* = 0.003), indicating that early rsFC changes may predict subsequent treatment response at 4 weeks. Marginal associations were observed between rsFC changes and concurrent symptom improvements at both the 2- and 8-week intervals (rho = -0.303, *p* = 0.098; rho = -0.292, *p* = 0.072). No significant correlations were found between rsFC changes at the 2-week time point and HAMD score reductions at the 8-week time point (rho = -0.273, *p* = 0.137), nor between baseline rsFC and baseline HAMD scores (rho = -0.049, *p* = 0.751) (Figure S3a-c).

**A brief commentary on the implications of supplementary findings**

In the supplementary analyses, we observed that rsFC between the right amygdala and the left SMA/mSFG was lower in the MDDr group but higher in the MDDnr group compared with HCs at baseline. Furthermore, this rsFC exhibited distinct longitudinal trajectories in the MDDr and MDDnr groups during treatment. Notably, this rsFC normalized after 2 weeks of treatment and persisted through 8 weeks. Correlation analyses further indicated that both baseline rsFC and its early changes (2 weeks) were associated with treatment response, and that longitudinal changes in rsFC were aligned with symptom improvement, although some correlations did not survive stringentmultiple comparisons correction. These findings provide additional insights into the neural mechanisms underlying antidepressant response and the basis of interindividual variability in treatment efficacy. Compared with findings involving the right basolateral (BL) amygdala, the spatial localization of SMA differed (e.g., right vs. left). This discrepancy may reflect differences between connectivity patterns derived from the right BL amygdala and those derived from the right whole amygdala. We speculated that other amygdala subregions may contribute to this divergence, although no significant effects were detected in the other two subregions separately. Future studies with larger samples are warranted to validate this speculation.

**Section 2 Sensitivity analyses**

**rsFC between the right BL amygdala and the right SMA**

A significant group × time interaction was observed in rsFC between the right BL amygdala and the right SMA (F = 6.564, *p* = 0.003, ηp² = 0.19; Figure S4 and Table S6), indicating distinct longitudinal trajectories in the MDDr and MDDnr groups. Simple effects analyses showed that in the MDDr group, rsFC increased from baseline to 2-week time point (t = -3.524, *p*_FDR_ = 0.003, Cohen’s d = -1.288), and showed a trend toward an increase from baseline to 8-week time point (t = -1.995, *p*_FDR_ = 0.076, Cohen’s d = -0.732), with no significant change between 2- and 8-week time points (t = 1.520, *p*_FDR_ = 0.134, Cohen’s d = 0.556). In contrast, no significant changes in rsFC were observed across any time intervals in the MDDnr group (baseline to the 2-week time point: t = 1.596, *p*_FDR_ = 0.349, Cohen’s d = 0.583; baseline to the 8-week time point: t = 0.458, *p*_FDR_ = 0.649, Cohen’s d = 0.167; 2- to 8-week time points: t = -1.138, *p*_FDR_ = 0.390, Cohen’s d = -0.415). At baseline, rsFC was lower in the MDDr group than in the MDDnr group (t = -2.240, *p* = 0.028, Cohen’s d = -0.886), but higher at 2-week time point (t = 2.481, *p* = 0.015, Cohen’s d = 0.985), with no significant difference observed at 8-week time point (t = 0.035, *p* = 0.972, Cohen’s d = 0.014).

No significant main effect of group or time were observed in this rsFC (F = 0.021, *p* = 0.886, ηp² <0.001; F = 1.042, *p* = 0.360, ηp² = 0.04).

**Effects of antidepressant treatment on the identified rsFC of the right BL amygdala in lorazepam or non-lorazepam subgroup**

In this study, 18 MDDr and 12 MDDnr did not receive lorazepam, whereas 7 MDDr and 11 MDDnr received lorazepam.

In the lorazepam subgroup, no significant group × time interaction was observed in rsFC between the right BL amygdala and the right SMA (F = 2.219, *p* = 0.127, ηp² = 0.13). No significant main effect of group or time were observed in this rsFC (F = 1.504, *p* = 0.243, ηp² = 0.11; F = 2.483, *p* = 0.101, ηp² = 0.15).

In the non-lorazepam subgroup, a significant group × time interaction was observed in rsFC between the right BL amygdala and the right SMA (F = 4.301, *p* = 0.019, ηp² = 0.15; Figure S5 and Table S7), indicating distinct longitudinal trajectories in the MDDr and MDDnr groups. Simple effects analyses showed that in the MDDr group, rsFC increased from baseline to 2- or 8-week time points (t = -3.529, *p*_FDR_ = 0.003, Cohen’s d = -1.280; t = -2.802, *p*_FDR_ = 0.011, Cohen’s d = -0.977), with no significant change between the 2- and 8-week time points (t = 0.808, *p*_FDR_ = 0.423, Cohen’s d = 0.303). In contrast,, no significant changes were observed across any time intervals in the MDDnr group (baseline to 2-week time point: t = 0.830, *p*_FDR_ = 0.755, Cohen’s d = 0.412; baseline to 8-week time point: t = 0.314, *p*_FDR_ = 0.755, Cohen’s d = 0.135; 2- to 8-week time points: t = -0.559, *p*_FDR_ = 0.755, Cohen’s d = -0.278). At baseline, rsFC was significantly lower in the MDDr group than in the MDDnr group (t = -2.575, *p* = 0.012, Cohen’s d = -1.084), whereas no group differences were observed at 2- or 8-week time points (t = 1.163, *p* = 0.249, Cohen’s d = 0.608; t = 0.063, *p* = 0.950, Cohen’s d = 0.028).

No significant main effect of group or time were observed in this rsFC (F = 0.231, *p* = 0.635, ηp² = 0.009; F = 1.482, *p* = 0.237, ηp² = 0.06).

Table S1 Group difference in rsFC of the right BL amygdala at baseline.

| **Seeded region** | **Hemisphere** | **Area** | **BA** | **Cluster size** | **MNI coordinates** | **Peak F values** | **Cluster-level *p*_FWE_** |
| --- | --- | --- | --- | --- | --- | --- | --- |
| Right BL amygdala | Right | SMA | 6 | 105 | 6 -22 62 | 11.64 | 0.041 |

Abbreviations: BL amygdala: basolateral amygdala; SMA: supplementary motor area.

Table S2 Comparisons of rsFC between the right BL amygdala and the right SMA in MDDr and MDDnr groups versus HCs at 2-week and 8-week time points.

| **Time** | **b^1^** | **SE^1^** | **t^1^** | ***p*^1^** | **b^2^** | **SE^2^** | **t^2^** | ***p*^2^** |
| --- | --- | --- | --- | --- | --- | --- | --- | --- |
| **2-week** | 0.014 | 0.040 | 0.350 | 0.728 | -0.050 | 0.041 | -1.223 | 0.227 |
| **8-week** | 0.000 | 0.035 | -0.012 | 0.990 | 0.004 | 0.033 | 0.133 | 0.895 |

Notes: 1: MDDr versus HC; 2: MDDnr versus HC; HC: available only at baseline.

Table S3 Group differences in rsFC of the right amygdala at baseline.

| **Seeded region** | **Hemisphere** | **Area** | **BA** | **Cluster size** | **MNI coordinates** | **Peak F values** | **Cluster-level *p*_FWE_** |
| --- | --- | --- | --- | --- | --- | --- | --- |
| Right amygdala | Left | SMA/mSFG | 6/8 | 199 | -6 16 56 | 16.54 | 0.001 |

Abbreviations: SMA: supplementary motor area; mSFG: medial superior frontal gyrus.

Table S4 The interaction effect, group effect and time effect in rsFC of the right amygdala.

| **RsFc** | | **MDDr_0** | **MDDr_2** | **MDDr_8** | **MDDnr_0** | **MDDnr_2** | **MDDnr_8** | **Interaction**  **F/*p*/ηp²** | **Group**  **F/*p*/ηp²** | **Time**  **F/*p*/ηp²** |
| --- | --- | --- | --- | --- | --- | --- | --- | --- | --- | --- |
| **Right amygdala-L.SMA/mSFG** | -0.08 ± 0.03 | | 0.02 ± 0.03 | -0.05 ± 0.03 | 0.13 ± 0.03 | 0.05 ± 0.04 | 0.07 ± 0.03 | 4.559/0.013/0.10 | 16.523/< 0.001/0.27 | 0.218/0.805/0.005 |

Notes: The functional connectivity values of different groups at different time points were represented by estimated marginal means ± standard error.

Abbreviations: 0: baseline; 2: 2-week of follow-up; 8: 8-week of follow-up; SMA: supplementary motor area; mSFG: medial superior frontal gyrus.

Table S5 Comparisons of rsFC between the right amygdala and the left SMA/mSFG in MDDr and MDDnr groups versus HCs at 2-week and 8-week time points.

| **Time** | **b^1^** | **SE^1^** | **t^1^** | ***p*^1^** | **b^2^** | **SE^2^** | **t^2^** | ***p*^2^** |
| --- | --- | --- | --- | --- | --- | --- | --- | --- |
| **2-week** | -0.038 | 0.037 | -1.049 | 0.299 | 0.024 | 0.040 | 0.600 | 0.551 |
| **8-week** | -0.067 | 0.037 | -1.783 | 0.080 | 0.046 | 0.033 | 1.411 | 0.163 |

Notes: 1: MDDr versus HC; 2: MDDnr versus HC; HC: available only at baseline.

Table S6 The interaction effect, group effect and time effect in rsFC of the right BL amygdala.

| **RsFc** | | **MDDr_0** | **MDDr_2** | **MDDr_8** | **MDDnr_0** | **MDDnr_2** | **MDDnr_8** | **Interaction**  **F/*p*/ηp²** | **Group**  **F/*p*/ηp²** | **Time**  **F/*p*/ηp²** |
| --- | --- | --- | --- | --- | --- | --- | --- | --- | --- | --- |
| **Right BL amygdala-R.SMA** | 0.01 ± 0.04 | | 0.18 ± 0.04 | 0.11 ± 0.04 | 0.13 ± 0.04 | 0.05 ± 0.04 | 0.11 ± 0.04 | 6.564/0.003/0.19 | 0.021/0.886/<0.001 | 1.042/0.360/0.04 |

Notes: The functional connectivity values of different groups at different time points were represented by estimated marginal means ± standard error.

Abbreviations: 0: baseline; 2: 2-week of follow-up; 8: 8-week of follow-up; BL amygdala: basolateral amygdala; SMA: supplementary motor area.

Table S7 The interaction effect, group effect and time effect in rsFC of the right BL amygdala in the non-lorazepam subgroup.

| **RsFc** | | **MDDr_0** | **MDDr_2** | **MDDr_8** | **MDDnr_0** | **MDDnr_2** | **MDDnr_8** | **Interaction**  **F/*p*/ηp²** | **Group**  **F/*p*/ηp²** | **Time**  **F/*p*/ηp²** |
| --- | --- | --- | --- | --- | --- | --- | --- | --- | --- | --- |
| **Right BL amygdala-R.SMA** | 0.00 ± 0.12 | | 0.18 ± 0.18 | 0.14 ± 0.10 | 0.13 ± 0.15 | 0.07 ± 0.19 | 0.12 ± 0.14 | 4.301/0.019/0.15 | 0.231/0.635/0.009 | 1.482/0.237/0.06 |

Notes: The functional connectivity values of different groups at different time points were represented by estimated marginal means ± standard error.

Abbreviations: 0: baseline; 2: 2-week of follow-up; 8: 8-week of follow-up; BL amygdala: basolateral amygdala; SMA: supplementary motor area.


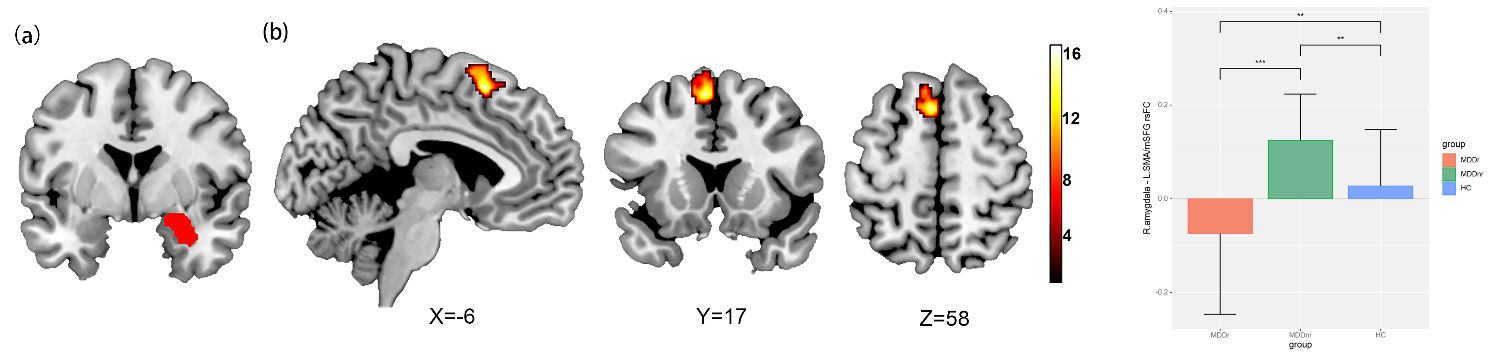


**Figure S1** Differences in baseline rsFC among the MDDr, MDDnr, and HC groups. (a) Seed region located in the right amygdala. (b) Group differences in baseline rsFC between the right amygdala and the left SMA/mSFG. ** *P* < 0.01, *** *P* < 0.001. SMA: supplementary motor area; mSFG: medial superior frontal gyrus.


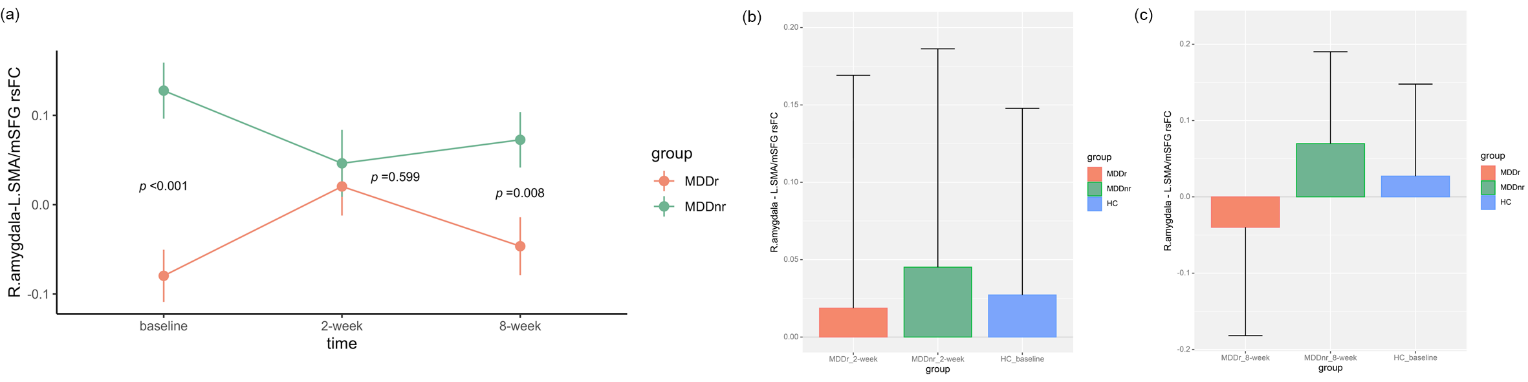


**Figure** **S2** Longitudinal analysis of the identified rsFC in the MDDr and MDDnr groups: (a) Significant group × time interaction effect in rsFC between the right amygdala and the left SMA/mSFG. (b) Comparisons of rsFC between the right amygdala and the left SMA/mSFG at 2-week time point between patients (MDDr or MDDnr) and HCs (available only at baseline). (c) Comparisons of rsFC between the right amygdala and the left SMA/mSFG at 8-week time point between patients (MDDr or MDDnr) and HCs (available only at baseline). SMA: supplementary motor area; mSFG: medial superior frontal gyrus.


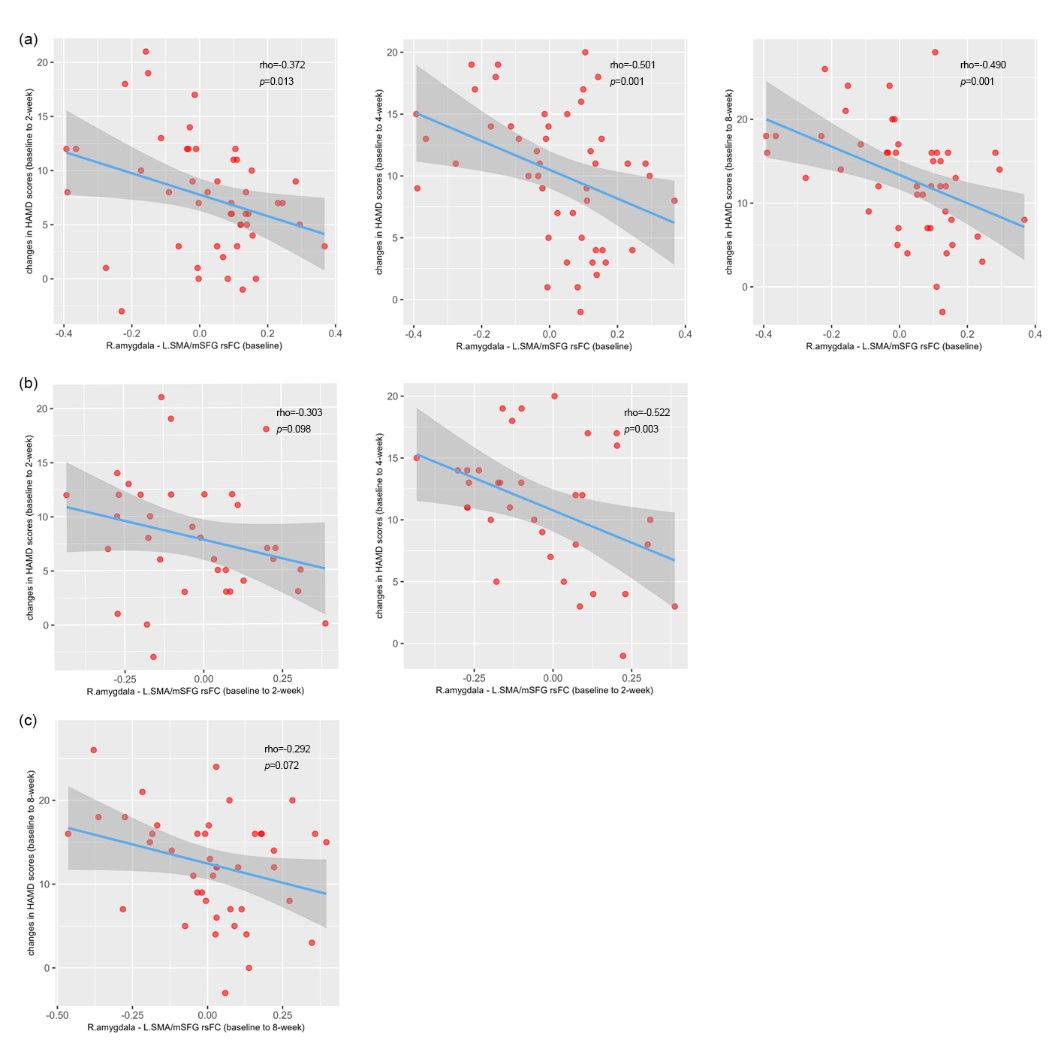


**Figure S3** Clinical correlates of the identified rsFC and their changes. (a) Clinical correlates of the rsFC between the right amygdala and the left SMA/mSFG at baseline. (b) Clinical correlates of the rsFC between the right amygdala and the left SMA/mSFG from baseline to 2-week time point. (c) Clinical correlate of the rsFC between the right amygdala and the left SMA/mSFG from baseline to 8-week time point. SMA: supplementary motor area; mSFG: medial superior frontal gyrus.


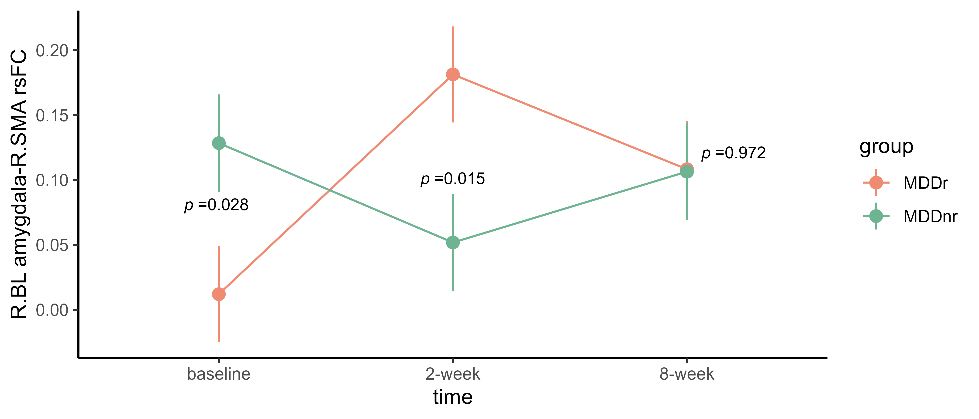


**Figure S4** Longitudinal analysis of the identified rsFC in the MDDr and MDDnr groups: Significant group × time interaction effect in rsFC between the right BL amygdala and the right SMA. BL amygdala: basolateral amygdala; SMA: supplementary motor area.


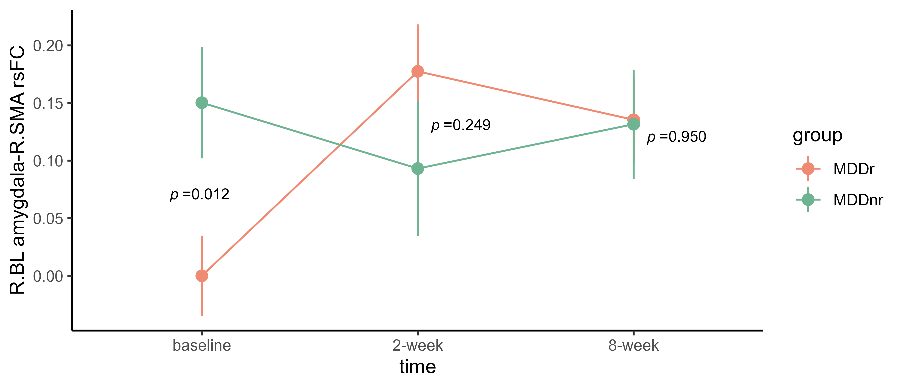


**Figure S5** Longitudinal analysis of the identified rsFC in the MDDr and MDDnr groups: (a) Significant group × time interaction effect in rsFC between the right BL amygdala and the right SMA. BL amygdala: basolateral amygdala; SMA: supplementary motor area.
